# Modeling Radiologists’ Assessments to Explore Pairing Strategies for Optimized Double Reading of Screening Mammograms

**DOI:** 10.1177/0272989X241264572

**Published:** 2024-07-30

**Authors:** Jessie J. J. Gommers, Craig K. Abbey, Fredrik Strand, Sian Taylor-Phillips, David J. Jenkinson, Marthe Larsen, Solveig Hofvind, Mireille J. M. Broeders, Ioannis Sechopoulos

**Affiliations:** Department of Medical Imaging, Radboud University Medical Center, Nijmegen, The Netherlands; Department of Psychological and Brain Sciences, University of California, Santa Barbara, CA, USA; Department of Oncology-Pathology, Karolinska Institute, Stockholm, Sweden; Breast Radiology, Karolinska University Hospital, Stockholm, Sweden; Warwick Medical School, University of Warwick, Coventry, UK; Warwick Medical School, University of Warwick, Coventry, UK; Section for Breast Cancer Screening, Cancer Registry of Norway, Oslo, Norway; Section for Breast Cancer Screening, Cancer Registry of Norway, Oslo, Norway; Department of Health and Care Sciences, UiT The Arctic University of Norway, Tromsø, Norway; Dutch Expert Center for Screening (LRCB), Nijmegen, The Netherlands; IQ Health Science Department, Radboud University Medical Center, Nijmegen, The Netherlands; Department of Medical Imaging, Radboud University Medical Center, Nijmegen, The Netherlands; Dutch Expert Center for Screening (LRCB), Nijmegen, The Netherlands; Technical Medicine Center, University of Twente, Enschede, The Netherlands

**Keywords:** breast cancer, screening, double reading, modeling, optimization

## Abstract

**Purpose:**

To develop a model that simulates radiologist assessments and use it to explore whether pairing readers based on their individual performance characteristics could optimize screening performance.

**Methods:**

Logistic regression models were designed and used to model individual radiologist assessments. For model evaluation, model-predicted individual performance metrics and paired disagreement rates were compared against the observed data using Pearson correlation coefficients. The logistic regression models were subsequently used to simulate different screening programs with reader pairing based on individual true-positive rates (TPR) and/or false-positive rates (FPR). For this, retrospective results from breast cancer screening programs employing double reading in Sweden, England, and Norway were used. Outcomes of random pairing were compared against those composed of readers with similar and opposite TPRs/FPRs, with positive assessments defined by either reader flagging an examination as abnormal.

**Results:**

The analysis data sets consisted of 936,621 (Sweden), 435,281 (England), and 1,820,053 (Norway) examinations. There was good agreement between the model-predicted and observed radiologists’ TPR and FPR (*r* ≥ 0.969). Model-predicted negative-case disagreement rates showed high correlations (*r* ≥ 0.709), whereas positive-case disagreement rates had lower correlation levels due to sparse data (*r* ≥ 0.532). Pairing radiologists with similar FPR characteristics (Sweden: 4.50% [95% confidence interval: 4.46%–4.54%], England: 5.51% [5.47%–5.56%], Norway: 8.03% [7.99%–8.07%]) resulted in significantly lower FPR than with random pairing (Sweden: 4.74% [4.70%–4.78%], England: 5.76% [5.71%–5.80%], Norway: 8.30% [8.26%–8.34%]), reducing examinations sent to consensus/arbitration while the TPR did not change significantly. Other pairing strategies resulted in equal or worse performance than random pairing.

**Conclusions:**

Logistic regression models accurately predicted screening mammography assessments and helped explore different radiologist pairing strategies. Pairing readers with similar modeled FPR characteristics reduced the number of examinations unnecessarily sent to consensus/arbitration without significantly compromising the TPR.

**Highlights:**

- A logistic-regression model can be derived that accurately predicts individual and paired reader performance during mammography screening reading.
- Pairing screening mammography radiologists with similar false-positive characteristics reduced false-positive rates with no significant loss in true positives and may reduce the number of examinations unnecessarily sent to consensus/arbitration.

## Graphical abstract

**Graphical Abstract:**
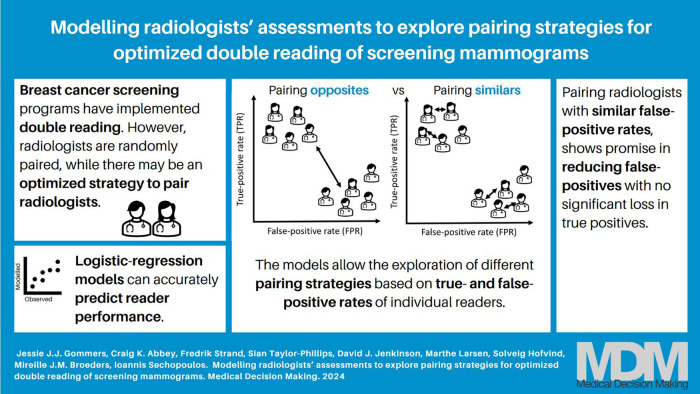
This is a visual representation of the abstract.

Breast cancer remains a major global health concern, and early detection plays a crucial role in improving patient outcomes. Population-based mammography screening programs have shown to be effective in reducing breast cancer–related mortality due to earlier detection of the disease.^1–4^ However, the challenges posed by the complexity of mammographic images and the potential for human error underline the need for continuous efforts to improve the interpretation of screening mammography. One approach to improving mammography interpretation is the implementation of double reading. Screening programs in Europe, Australia, and New Zealand have implemented double reading, in which each screening examination is assessed by 2 independent readers, increasing the cancer detection rate (CDR) for combined assessments.^5–7^ Usually, discordant assessments between the 2 readers are referred to an arbitrator or discussed at a consensus meeting, so that a final recall decision can be made. In some screening programs, concordant positive assessments are also referred for a final check by an arbitrator or by consensus. In countries in which screening mammograms are single read, a potential double-reading strategy is to use artificial intelligence (AI) as a second independent reader.^8–11^

The success of double reading, however, may rely on how the radiologists are paired. Countries currently employing double reading generally pair readers randomly without considering the characteristics of the readers. However, matching radiologists with different strengths can potentially maximize the detection of breast cancer while minimizing the chances of false positives. A previously published study showed that the accuracy of mammography interpretation with double reading can indeed be improved by optimizing the set of paired radiologists.^12^ However, this study did not identify what the a priori pairing strategy should be to achieve this optimization. A potential pairing optimization strategy for screening may be based on individual reader performance characteristics, which vary considerably among radiologists.^13^ The present study was triggered by the findings of another previous study,^14^ which found that radiologist screening performance characteristics influenced the performance of radiologist pairs. However, that study was not able to detect significant variations in overall group performance resulting from different pairing strategies based on individual characteristics. This might be due to the limitation that with existing real-world screening data, each screening examination is read by only 2 readers, and therefore exhaustive exploration of different pairing strategies is not possible.

Previous studies have used mathematical models of the human observer, known as model observers, to investigate the diagnostic potential of different images and consequently the performance of radiologists.^15,16^ However, to the best of our knowledge, there are no studies that used retrospective screening data to model radiologist assessments and immediately use this for generating a new data set. While several studies have explored the association between radiologist characteristics and screening performance, they did not focus on modeling individual radiologists explicitly.^17,18^

Therefore, this study aims to develop an explanatory logistic regression model designed to simulate individual radiologists’ assessments and use it to investigate if there is a strategy for pairing radiologists based on individual screening performance characteristics that would optimize overall screening performance. By modeling radiologists’ assessments and creating interpretations involving all readers for all examinations, we are able to explore various radiologist pairing strategies against random pairing with sufficient statistical power.

## Methods

### Reader Model

#### Model definition

A logistic model is derived to fit observed data that is binary in nature. Let $Y^{-}$ and $Y^{+}$ represent the screening outcomes (1 = recall, 0 = return to screening) for negative (noncancer) and positive (cancer) cases, respectively. When $Y^{-}$ has a value of 1 for a given case and reader, the decision is defined as a false positive, and when $Y^{+}$ has a value of 1, the decision is defined as a true positive.

The model posits a latent decision variable for each reader and case. Let the readers be indexed by j=1,…,J , negative cases by $k=1,\ldots ,K_{j}^{-}$ , and positive cases by $k=1,\ldots ,K_{j}^{+}$ . Note that positive and negative cases are considered to be completely independent ( $K^{+}<<K^{-}$ ). The decision variables, $q_{\mathrm{jk}}^{-}$ for negative cases and $q_{\mathrm{jk}}^{+}$ for positive cases are assumed to be the sum of a reader-specific effect, $r_{j}^{-}$ or $r_{j}^{+}$ , and a case-specific effect, $C_{k}^{-}$ or $C_{k}^{+}$ ,

(1) $$q_{jk}^{-}=r_{j}^{-}+C_{k}^{-}\;\mathrm{and}\;q_{jk}^{+}=r_{j}^{+}+C_{k}^{+}.$$

The reader effects, parameterized by $r_{j}^{-}$ and $r_{j}^{+}$ , are considered to be fixed effects (and they include any global intercept parameter). The case effects, $C_{k}^{-}$ and $C_{k}^{+}$ , are assumed to be random effects, modeled as realizations of a normal random process with a mean of 0 and a standard deviation of $\sigma_{C^{-}}$ or $\sigma_{C^{+}}$ for the negative and positive examinations, respectively. A logistic link function converts the $q_{\mathrm{jk}}$ variables into abnormal-interpretation probabilities,

(2) $$a_{jk}^{-}=\frac{exp(q_{jk}^{-})}{1+exp(q_{jk}^{-})}\;\mathrm{and}\;a_{jk}^{+}=\frac{exp(q_{jk}^{+})}{1+exp(q_{jk}^{+})}.$$

Note that $a_{jk}^{-}$ represents the probability of a false-positive interpretation and $a_{\mathrm{jk}}^{+}$ represents a true-positive interpretation, conditioned on a specific reader and a specific case. For a given reader, the unconditional probability of an abnormal interpretation (across cases) is the expected value of the probabilities in equation 2 over the distribution of case effects,

(3) $$a_{j}^{-}=E{\left(a_{\mathrm{jk}}^{-}\right)}_{C_{k}^{-}\sim N\left(0,\sigma_{C^{-}}\right)}\;\mathrm{and}\;a_{j}^{+}=E{\left(a_{\mathrm{jk}}^{+}\right)}_{C_{k}^{+}\sim N\left(0,\sigma_{C^{+}}\right)}.$$

These probabilities represent the reader’s false-positive rate (1 − specificity) and true-positive rate (sensitivity) over the population of cases. Let $A_{j}^{-}$ and $A_{j}^{+}$ represent the number of abnormal findings for negative and positive cases, respectively; these are presumed to be related to the reader parameters through binomial distributions,

(4) $$A_{j}^{-}\sim B\left(a_{j}^{-},K_{j}^{-}\right)\;\mathrm{and}\;A_{j}^{+}\sim B\left(a_{j}^{+},K_{j}^{+}\right).$$

Equations 1 to 4 relate the model parameters (equation 1) to observed single-reader data (equation 4).

Up to this point, we have analyzed only single-reader performance, but we can use a similar approach to extend the approach to disagreement rates between paired readers. For a pair of readers, indexed by j and j′ ( j≠j′ ), let the negative cases be indexed by $k=1,\ldots ,K_{jj^{\prime }}^{-}$ and the positive cases be indexed by $k=1,\ldots ,K_{jj^{\prime }}^{+}$ . We can define case-specific disagreement rates based on the probabilities in equation 2 as

(5) $$d_{jj^{\prime }k}^{-}=a_{\mathrm{jk}}^{-}\left(1-a_{j^{\prime }k}^{-}\right)+a_{j^{\prime }k}^{-}\left(1-a_{\mathrm{jk}}^{-}\right)\;\mathrm{and}\;d_{jj^{\prime }k}^{+}=a_{\mathrm{jk}}^{+}\left(1-a_{j^{\prime }k}^{+}\right)+a_{j^{\prime }k}^{+}\left(1-a_{\mathrm{jk}}^{+}\right).$$

These case-specific probabilities can be marginalized into reader-pair–specific probabilities by taking the expectation across cases,

(6) $$d_{jj^{\prime }}^{-}=E{\left(d_{jj^{\prime }k}^{-}\right)}_{C_{k}^{-}\sim N\left(0,\sigma_{C^{-}}\right)}\;\mathrm{and}\;d_{jj^{\prime }}^{+}=E{\left(d_{jj^{\prime }k}^{+}\right)}_{C_{k}^{+}\sim N\left(0,\sigma_{C^{+}}\right)}.$$

Let $D_{jj\prime }^{-}$ and $D_{jj^{\prime }}^{+}$ represent the number of disagreements for negative and positive cases, respectively; these are presumed to be related to the reader parameters through binomial distributions,

(7) $$D_{jj^{\prime }}^{-}\sim B\left(d_{jj^{\prime }}^{-},K_{jj^{\prime }}^{-}\right)\;\mathrm{and}\;D_{jj^{\prime }}^{+}\sim B\left(d_{jj^{\prime }}^{+},K_{jj^{\prime }}^{+}\right).$$

Note that both the abnormal-interpretation probabilities, $a_{j}^{-}$ and $a_{j}^{+}$ , and the disagreement probabilities, $d_{jj^{\prime }}^{-}$ and $d_{jj^{\prime }}^{+}$ , are related to the data through binomial distributions. But the disagreement component of the model plays a crucial role, since the model is not identifiable (i.e., multiple parameter values produce identical abnormal-interpretation probabilities) based on the abnormal interpretation component alone.

#### Model fitting

Our implementation of the model computed expectations in equations 3 and 6 using Monte Carlo integration with 1,000,000 sample case effects for each integral. This resulted in 1,000,000 sample abnormal interpretation and disagreement probabilities in equations 2 and 5, and these were averaged to get high-precision estimates of reader abnormal-interpretation probabilities and paired disagreement rates. For rates that were near the estimated values, the Monte Carlo coefficient of variation on abnormal interpretation rates was less than 1%.

Estimation of the reader parameters and the case variances was performed by maximizing the log-likelihood defined by the binomial distributions in equations 4 and 7. This approach allows the computation of individual reader performance characteristics as well as paired disagreement probabilities. We describe this for positive cases, but there is a corresponding log-likelihood for negative cases. The resulting optimization function is

(8) $$\lambda \left(r_{1}^{+},\ldots ,r_{N_{R}}^{+},\sigma_{C^{+}}\right)=\sum_{j=1}^{N_{R}}(K_{j}^{+}\ln\left(a_{j}^{+}\right)+\left(K_{j}^{+}-A_{j}^{+}\right)\ln\left(1-a_{j}^{+}\right))+\sum_{\left(j,j\prime \right)\in \Pi }(K_{jj^{\prime }}^{+}\ln\left(d_{jj^{\prime }}^{+}\right)+\left(K_{jj^{\prime }}^{+}-D_{jj^{\prime }}^{+}\right)\ln\left(1-d_{jj^{\prime }}^{+}\right))+X.$$

where X represents constant terms unrelated to the parameters (log-factorial components) and Π represents the set of observed reader pairs in the data. An optimization algorithm implementing Powell’s method^19^ with standard convergence criterion ( $f_{\mathrm{Tol}}=10^{-4}$ ) was used to find maximum likelihood estimates of the reader parameters ( $r_{j}^{-}$ and $r_{j}^{+}$ ) and case standard deviations ( $\sigma_{C^{-}}$ and $\sigma_{C^{+}}$ ) that maximize this function.

For graphical evaluations of model fit, estimated reader abnormal interpretation rates ( $a_{j}^{-}$ and $a_{j}^{+}$ , equation 3) and paired disagreement rates ( $d_{jj^{\prime }}^{-}$ and $d_{jj^{\prime }}^{+}$ , equation 6) were compared to direct estimates of the quantities from the data. Direct estimates for the abnormal interpretation rates are given by

(9) $$\mathrm{FP}R_{j}=A_{j}^{-}/K_{j}^{-}\;\mathrm{and}\;\mathrm{TP}R_{j}=A_{j}^{+}/K_{j}^{+}.$$

and for the disagreement rates by

(10) $${\mathrm{DR}}_{jj^{\prime }}^{-}=D_{jj^{\prime }}^{-}/K_{jj^{\prime }}^{-}\;\mathrm{and}\;{\mathrm{DR}}_{jj^{\prime }}^{+}=D_{jj^{\prime }}^{+}/K_{jj^{\prime }}^{+}.$$

These rates were adjusted using the Agresti-Coull procedure,^20^ which was also used to obtain 95% confidence intervals.

### Double Reading Simulation

Assuming the logistic regression models adequately accounted for single-reader performance and disagreement rates between paired readers, the reader parameters ( $r_{j}^{-}$ and $r_{j}^{+}$ ) and case standard deviations ( $\sigma_{C^{-}}$ and $\sigma_{C^{+}}$ ) were used to simulate double reading and investigate the effect of different pairings of readers computationally. A random pairing strategy was compared against 2 main other pairing strategies, including 1) pairing readers with *similar* performance characteristics and 2) pairing readers with *opposite* performance characteristics. In total, 7 different pairing strategies were simulated:

1. Similar true-positive rate (TPR)
2. Similar false-positive rate (FPR)
3. Opposite TPR
4. Opposite FPR
5. Total similars = combination of TPR and FPR (TPR + FPR × slope)
6. Total opposites = combination of TPR and FPR (TPR + FPR × slope)
7. Random

**Figure 1:**
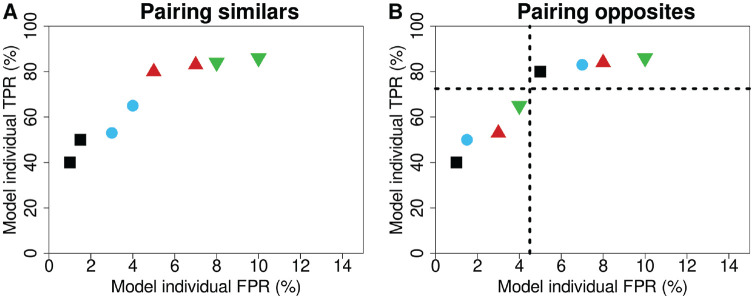
Explanation for the pairing of readers being either (A) similar or (B) opposite in their performance characteristics. The colors and symbols represent the pairs of readers, and the dashed lines are the median TPR and FPR of the readers. FPR, false-positive rate; TPR, true-positive rate.

For the pairing of similar and opposite readers, the modeled reader-specific TPR and FPR from equation 3 were used. For the 3 similar pairing strategies, the available reader with the lowest performance value in question (TPR, FPR, TPR + FPR × slope) was selected and paired with the reader closest in corresponding performance (i.e., had the second lowest value, Figure 1A). This pairing continued for the readers with the third and fourth lowest values, and so forth, until all readers were assigned. For the 3 opposite pairing strategies, readers were split into 2 groups, as above and below the median of that performance metric. The available reader with the lowest performance value in question (TPR, FPR, TPR + FPR × slope) in one group (i.e., below the median) was paired with the available reader with the lowest value in the other group (i.e., above the median), until all readers were assigned (Figure 1B). For the total similar and opposite pairing strategies, the same pairing strategy as shown in Figure 1A and B was used, but now the slopes of the linear regression lines of the individual TPR and FPR were used for pairing (TPR + FPR × slope). For random pairing, readers were randomly sampled without replacement, making sure that no reader was paired with themselves.

For pairs, case-specific paired abnormal interpretation rates based on the probabilities in equation 2 are defined as

(11) $$p_{jj^{\prime }(k)}^{-}=a_{\mathrm{jk}}^{-}+a_{j^{\prime }k}^{-}-(a_{\mathrm{jk}}^{-})(a_{j^{\prime }k}^{-})\;\;\mathrm{and}\;p_{jj^{\prime }(k)}^{+}=a_{\mathrm{jk}}^{+}+a_{j^{\prime }k}^{+}-(a_{\mathrm{jk}}^{+})(a_{j^{\prime }k}^{+}).$$

where jj′(k) is defined as the pair of readers assigned a given examination. Equation 11 defines disagreement between 2 readers as an abnormal interpretation for the pair. This maintains a high sensitivity in which cancer cases are, at least, being discussed in a consensus meeting or send to a third reader for arbitration.^21^

### Data Sets

**Figure 2:**
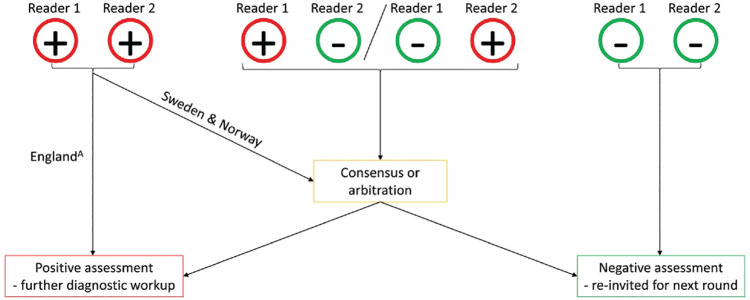
Explanation of the recall procedures in the involved data sets. The red circle (+) represents a positive assessment warranting further diagnostic workup, while the green circle (–) represents a negative assessment. ^A^At most centers, only discrepant readings were resolved with a third reader or consensus meeting. However, at certain centers with elevated recall rates, arbitration was also applied when both readers had a positive assessment.

For this study, the above-described model was derived using the screening reading results from 3 different breast cancer screening programs: the Swedish CSAW (Cohort of Screen-Age Women) data set,^22^ the English CO-OPS (Changing case Order to Optimize patterns of Performance in Screening) data set,^23,24^ and a data set from BreastScreen Norway. These data sets were also used in a previously published article that investigated pairing strategies based on reader performance.^14^ However, the previous publication included analysis performed on the observed data, rather than using the data to derive a reader model to exhaustively explore all possible pairings. The study population and screening procedures of the 3 data sets have been previously described.^14,22–26^ Briefly, the CSAW data set consists of women aged 40 to 74 y who participated in 2-view digital mammographic screening in the Stockholm region in Sweden between 2008 and 2015. The regional Ethical Review Board in Sweden approved the use of the CSAW data and waived the need for informed consent. The CO-OPS data set consists of women aged 47 to 73 y who attended 2-view digital mammography screening between 2012 and 2014 in England. Institutional Review Board approval for the original CO-OPS trial was obtained from Coventry and Warwickshire National Health Service Research Ethics Committee (June 27, 2012), and each breast screening director gave informed consent. The nationwide data set from the Norwegian screening program consists of women aged 50 to 69 y who underwent 2-view digital screening mammography between 2004 and 2018. The Norwegian data were obtained in accordance with the legal bases outlined in the Norwegian Cancer Registry Regulations of December 21, 2001, No. 47, and the requirement for informed consent was waived. In the 3 screening programs, mammograms are double read by 2 readers, who have access to prior mammograms if available. There are some variations in recall protocols, as depicted in Figure 2. Across all programs, agreements on negative assessments are deemed normal, while discrepancies are resolved either through a third reader’s evaluation or a consensus meeting. Notably, in Sweden and Norway, agreed positive assessments prompt discussion in a consensus meeting to assess the necessity of further diagnostic evaluation, whereas in England, such agreements typically lead to immediate recall.

Breast cancers included invasive cancers or ductal carcinoma in situ that were diagnosed with needle biopsy or surgery after diagnostic workup in screening or clinically in between screening rounds (i.e., interval cancer). Entries from symptomatic women and readings with missing reader data or inadequate images were excluded. For an appropriate fit of the logistic regression model, readers had to have at least 1 true-positive and false-positive assessment, and the number of true- and false-positives should not be equal to the total number of positive and negative examinations, respectively. To meet these criteria, pairs with a relatively low volume in the data set were also excluded.

### Simulation to Optimize Screening Performance

#### Double reading strategies

The logistic regression models were used to explore radiologist pairing strategies based on individual screening performance, as described above. To mimic real-world breast cancer screening, each of the 7 above-described pairing strategies was simulated by sampling 365 screening days with 4,000 examinations distributed over 32 batches per day. For each screening day, a random set of 16 readers was chosen. Within the simulations, each of the batches was read by 2 of the 16 randomly selected radiologists reading that day, with the constraint that each reader read about the same number of batches. Each day, 8 unique pairs were created, ensuring a similar number of pairs per day for all pairing strategies. All 7 simulations involved the same examinations and readers; the only difference was the pairing of the readers. In the end, all 7 simulations resulted in 1,460,000 examinations (4,000 × 365) with each having 2 individual outcome probabilities (reader 1 and reader 2) from equation 2 and a paired probability from equation 11. The main endpoint was the expected group TPR and FPR of each pairing strategy, given by

(12) $$\mathrm{FP}R_{\mathrm{total}}=\sum_{k^{-}=1}^{K^{-}}p_{jj^{\prime }(k^{-})}^{-}/K^{-}\;\mathrm{and}\;\mathrm{TP}R_{\mathrm{total}}=\sum_{k^{+}=1}^{K^{+}}p_{jj^{\prime }(k^{+})}^{+}/K^{+}.$$

Bootstrap resampling (*n* = 1,000) was used to obtain 95% confidence intervals for the group performance. The 95% confidence intervals were Bonferroni corrected for 6 comparisons and used to compare the 3 similar and 3 opposite pairing strategies against the random pairing strategy. TPR and FPR were plotted, and nonoverlapping confidence intervals were regarded as statistically significant.

In addition, differences in the number of true-positive (TP) and false-positive (FP) examinations between the different pairing strategies and the random pairing strategy were illustrated. For this, individual outcome probabilities (equation 2) were sampled as absolute values of 0 (indicating no suggested recall) or 1 (indicating suggested recall) according to the Bernoulli distribution, resulting in 3 potential paired assessments—concordant positive, concordant negative, and discordant assessments—facilitating the evaluation of differences in the number of TP and FP examinations.

#### Individual reading

To compare the performance of the double reading pairing strategies to that of individual readers, individual reader simulations were also performed. The simulations involved the same examinations and readers as for the paired simulations, but this time each examination was interpreted by only 1 of the 16 random readers reading that day, again with the constraint that each reader interpreted the same number of batches. Individual abnormal interpretation probabilities were obtained from equation 2 and used to calculate grouped TPR and FPR of examinations read by 1 reader only:

(13) $$\mathrm{FP}R_{\mathrm{single}}=\sum_{k^{-}=1}^{K^{-}}a_{j(k^{-})}^{-}/K^{-}\;\mathrm{and}\;\mathrm{TP}R_{\mathrm{single}}=\sum_{k^{+}=1}^{K^{+}}a_{j(k^{+})}^{+}/K^{+}.$$

where j(k) indicates the reader assigned to exam k . The 95% confidence intervals, obtained from bootstrap resampling (*n* = 1,000), were Bonferroni corrected for 7 comparisons contrasting the TPR and FPR of the 7 double reading strategies against the TPR and FPR of the individual reading.

All statistical analyses were performed in each of the 3 data sets separately. Model fitting was performed in the Interactive Data Language version 8.2.3 (IDL, L3Harris Geospatial, Broomfield, CO, USA), and the resulting model parameters were subsequently used to simulate pairing strategies in R studio version 4.1.0 (RStudio, PBC, Boston, MA, USA). The funding source ensured the authors’ independence in designing the study, interpreting the data, and writing the article.

## Results

**Figure 3:**
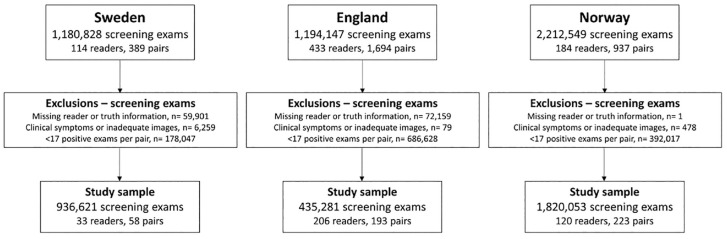
Flowchart of screening examinations after applying exclusion criteria.

Figure 3 summarizes how the final study samples were assembled. For an appropriate fit of our logistic regression model, an exclusion criterion of at least 17 positive reads per pair was needed to make sure that the number of true and false positives was not equal to the total number of positive and negative examinations, respectively. The final study samples consisted of *n* = 936,621 (Sweden), *n* = 435,281 (England), and *n* = 1,820,053 (Norway) screening examinations.

### Study Sample Characteristics

The population and reader characteristics varied among the study samples (Table 1). The Swedish study sample included the youngest population of women with a median age of 53 y at screening. Paired TPR and FPR in all study samples were higher than individual TPR and FPR as a result of our pairing rule, where disagreement between 2 readers was defined as a positive assessment. The English study sample showed the highest average individual TPR (78.2%) and paired TPR (83.0%), whereas the Norwegian study sample had the highest individual and paired FPRs (5.1% and 8.0%, respectively). Norway’s study sample also showed most disagreement between readers in a pair (5.6%).

**Table 1:** Population, Reader, and Pair Characteristics for the Study Samples after Selection Criteria

	Sweden	England	Norway
	*n* = 936,621 Examinations	*n* = 435,281 Examinations	*n* = 1,820,053 Examinations
Population characteristics			
Women, *n*	390,680	435,281	653,161
Median age at screening, years (IQR)	53 (46–62)	59 (53–65)^a^	59 (54–64)
Screening programs (double reading)			
Screening interval, mo	18–24^b^	36	24
Recalled examinations, per 1,000	17.8^c^	41.1	29.7
Detected cancers, per 1,000	3.3^c,d^	9.2^f^	5.8^f^
Interval cancers, per 1,000	1.8^c,e^	2.2^g^	1.8^g^
Reader characteristics			
Readers, *n*	33	206	120
Average true-positive rate, %	63.0	78.2	70.2
Average false-positive rate, %	3.5	4.1	5.1
Pair characteristics			
Pairs, *n*	58	193	223
Average true-positive rate, %	70.3	83.0	81.6
Average false-positive rate, %	5.0	5.5	8.0
Disagreement between readers in a pair, %	2.3	3.0	5.6

IQR, interquartile range.

a Age was missing for 4 screening examinations.

b Women aged 49 y and older were invited every 24 mo, while younger women were invited every 18 mo.

c The final screening assessment was missing for 16 screening examinations.

d Recall and breast cancer detection within 12 mo after screening.

e Women who did not have a screen-detected cancer but had a breast cancer diagnosed within 18 to 24 mo after screening.

f Breast cancer detected before the next screening examination as a result of recall at screening.

g Women who did not have a screen-detected cancer but had a breast cancer diagnosed before the next screening round.

### Model Fit

**Figure 4:**
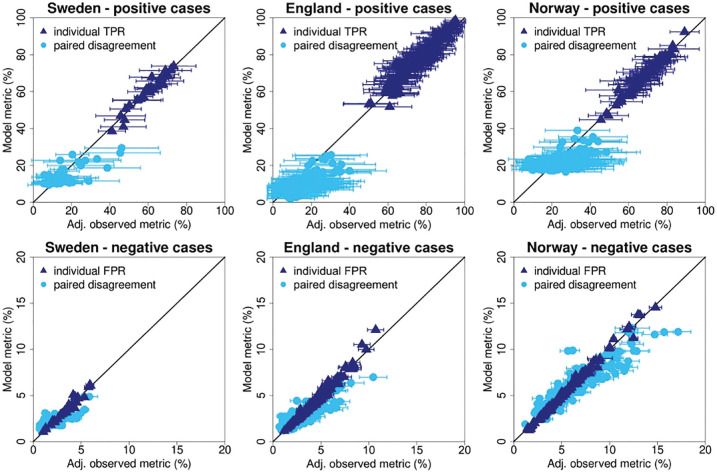
Comparison of modeled and observed single-reader performance and paired disagreement rates. The scatterplots show single-reader performance and paired disagreement rates for the Swedish, English, and Norwegian study sample. The dark-blue triangles represent single-reader performance (top: true-positive rates, bottom: false-positive rates). The light-blue dots represent the disagreement rates for pairs of readers (top: positive cases, bottom: negative cases). The diagonal represents perfect agreement between the modeled and observed data. The observed proportions were adjusted using the Agresti-Coull procedure for 95% confidence intervals. FPR, false-positive rate; TPR, true-positive rate.

The single-reader performance from the model showed good agreement with the Agresti-Coull adjusted observed data (Figure 4, dark-blue triangles). The individual TPR values showed Pearson correlation coefficients of 0.969, 0.973, and 0.988 for the Swedish, English, and Norwegian study sample, respectively. The individual FPR values had Pearson correlation coefficients of 0.978, 0.990 and 0.996 for the Swedish, English, and Norwegian study samples.

The light-blue dots in Figure 4 show the modeled disagreement rates compared against the observed data. For this comparison only, the pairs observed in the actual screening data set could be used (Sweden: 58 of the 528 possible pairs; England: 193 of the 21,115 possible pairs; Norway: 223 of the 7,140 possible pairs). Disagreement rates for positive cases showed lower levels of correlation with the Swedish, English, and Norwegian study samples, having Pearson correlation coefficients of 0.727, 0.658, and 0.532, respectively. As expected, pairs that read a relatively small number of examinations had large error bars for the estimates of positive pair disagreement. Disagreement rates for negative cases showed higher correlations with coefficients of 0.709, 0.846, and 0.923 for the Swedish, English, and Norwegian study samples, respectively.

**Figure 5:**
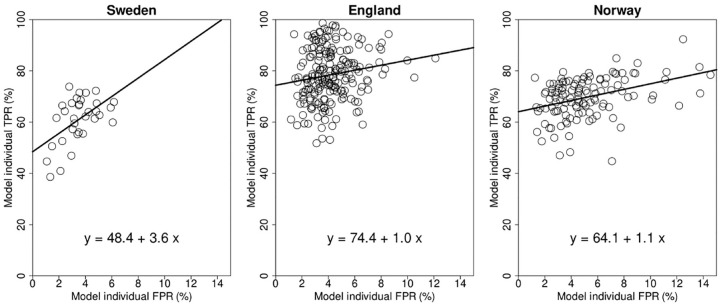
Modeled TPR and FPR of the individual readers. The scatterplots show true-positive and false-positive rates for the Swedish, English, and Norwegian study sample. The linear regression line shows the association between TPR and FPR. FPR, false-positive rate; TPR, true-positive rate.

The modeled individual TPR and FPR showed the commonly found association between TPR and FPR, with high TPR readers tending to have higher FPR (Figure 5).

### Screening Simulation

#### Double reading strategies

**Figure 6:**
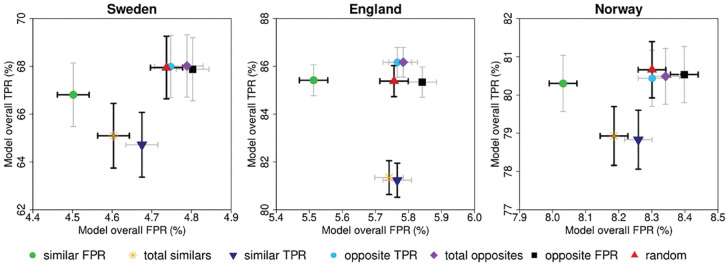
Screening performance for the different pairing strategies based on paired assessment. The scatterplots show overall true-positive and false-positive rates of the different pairing strategies for the Swedish, English, and Norwegian study samples. The colors and symbols represent the different pairing strategies. Error bars are 95% confidence intervals, obtained by bootstrap resampling (*n* = 1,000) and adjusted for 6 comparisons. Bold error bars indicate statistical significance. Please note that the axes are different, due to differences in TPR and FPR between the study samples. FPR, false-positive rate; TPR, true-positive rate.

The grouped TPR and FPR of the 7 modeled pairing strategies, based on the modeled individual TPR and FPR from Figure 5, are shown in Figure 6 and Table 2. The pattern for all 3 study samples looks similar. According to our pairing rule, pairing readers who are similar in a certain performance metric resulted in a lower value for the paired outcome of that metric. Pairing strategies involving readers with similar TPR (TPR Sweden: 64.72% [95% confidence interval {CI}: 63.36%–66.07%], England: 81.23% [95% CI: 80.52%–81.95%], Norway: 78.83% [95% CI: 78.06%–79.60%]) resulted in a statistically significant reduction of the TPR when compared with random pairing strategies (Sweden: 67.95% [95% CI: 66.64%–69.25%], England: 85.38% [95% CI: 84.73%–86.03%], Norway: 80.66% [95% CI: 79.93%–81.39%]). In addition, the FPR of pairing strategies involving 2 similar FPR readers (FPR Sweden: 4.50% [95% CI: 4.46%–4.54%], England: 5.51% [95% CI: 5.47%–5.56%], Norway: 8.03% [95% CI: 7.99%–8.07%]) was statistically significantly lower compared with the FPR of the random pairing strategies (Sweden: 4.74% [95% CI: 4.70%–4.78%], England: 5.76% [95% CI: 5.71%–5.80%], Norway: 8.30% [95% CI: 8.26%–8.34%]). Conversely, pairing opposite readers increased the value of the paired outcome of what they were opposite in. However, none of the pairing strategies resulted in a statistically significant increased TPR compared with the random pairing strategy (Figure 6, Table 2). To extrapolate these findings to the context of screening programs, the resulting recall rate (RR) and CDR of the different modeled pairing strategies were calculated (Figure A1). For all 3 data sets, pairing readers with similar FPR resulted in a significantly lower RR, while CDR did not significantly change.

**Table 2:** Screening Performance for the Different Pairing Strategies^a^

	Sweden		England		Norway	
	TPR (95% CI)	FPR (95% CI)	TPR (95% CI)	FPR (95% CI)	TPR (95% CI)	FPR (95% CI)
Similar FPR	66.81 (65.48–68.14)	4.50 (4.46–4.54)*	85.42 (84.77–86.07)	5.51 (5.47–5.56)*	80.30 (79.57–81.04)	8.03 (7.99–8.07)*
Total similars	65.10 (63.74–66.45)*	4.60 (4.56–4.64)*	81.35 (80.64–82.05)*	5.74 (5.70–5.78)	78.93 (78.16–79.70)*	8.19 (8.14–8.23)*
Similar TPR	64.72 (63.36–66.07)*	4.67 (4.63–4.72)	81.23 (80.52–81.95)*	5.77 (5.72–5.81)	78.83 (78.06–79.60)*	8.26 (8.22–8.30)
Opposite TPR	67.99 (66.69–69.29)	4.75 (4.71–4.79)	86.17 (85.54–86.79)	5.77 (5.72–5.81)	80.44 (79.70–81.17)	8.30 (8.26–8.34)
Total opposites	68.01 (66.71–69.32)	4.79 (4.75–4.83)	86.17 (85.55–86.79)	5.78 (5.74–5.83)	80.49 (79.76–81.22)	8.34 (8.30–8.38)
Opposite FPR	67.87 (66.55–69.19)	4.80 (4.76–4.84)	85.34 (84.70–85.97)	5.84 (5.80–5.89)	80.53 (79.80–81.27)	8.40 (8.36–8.44)*
Random (reference)	67.95 (66.64–69.25)	4.74 (4.70–4.78)	85.38 (84.73–86.03)	5.76 (5.71–5.80)	80.66 (79.93–81.39)	8.30 (8.26–8.34)

CI, confidence interval; FPR, false-positive rate; TPR, true-positive rate.

a TPR and FPR are percentages, and 95% CI are Bonferroni adjusted (*P* values <0.05/6) confidence intervals, obtained by bootstrap resampling (*n* = 1,000). Nonoverlapping CIs with the random pairing strategy were regarded as statistically significant, as indicated by the asterisk.

**Figure 7:**
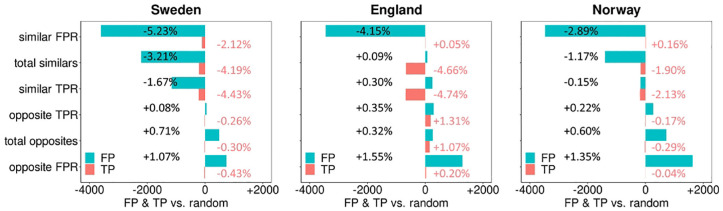
Change in the number of paired true-positives (pink) and false-positives (blue) for the different similar and opposite pairing strategies compared to the random pairing strategy. This simulation assumed a population size of 1,460,000 examinations. Positive assessments are determined by our pairing rule, which defines an examination as positive if any of the readers flags it as abnormal. Percentages indicate the change in the number of TP or FP compared with random pairing and vary as random pairing results in different numbers for TP and FP across the 3 different study samples. FP, false positive; TP, true positive.

Figure 7 shows the difference in the absolute number of TP and FP between the similar/opposite pairing strategies and the random pairing strategy, according to our pairing rule. The pairing strategy with similar FPR readers resulted in the best outcome, reducing the number of FPs with reductions of −3,605 (68,866→65,261; −5.23%), −3,447 (83,073→79,626; −4.15%), and −3,475 (120,146→116,671; −2.89%) FP screening examinations for Sweden, England, and Norway, respectively. The other pairing strategies resulted in similar or worse group outcomes. Compared with the random pairing strategy, the pairing strategy with similar TPR readers reduced the number of TP the most with −225 (5,084→4,859; −4.43%), −681 (14,364→13,683; −4.74%), and −192 (9,000→8,808; −2.13%) TP screening examinations for Sweden, England, and Norway, respectively. For the opposite pairing strategies, the number of FPs increased compared with random pairing, especially for pairs with opposite FPR characteristics readers (FP Sweden: [+739, 68,866→69,605; +1.07%], England: [+1,291, 83,073→84,364; +1.55%], Norway: [+1,627, 120,146→121,773; +1.35%]). The TPs for the opposite pairing strategies remained essentially similar.

#### Individual reading

**Figure 8:**
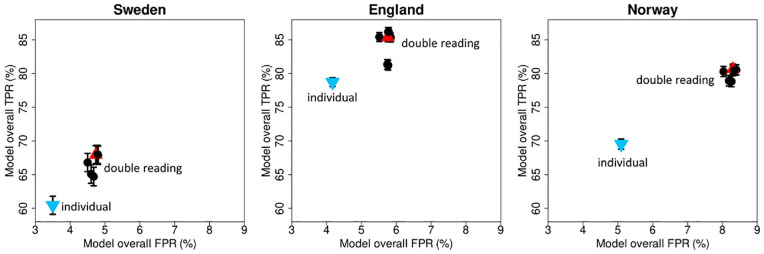
Screening performance for individual reading and different double reading strategies. The scatterplots show the overall true-positive and false-positive rates of the different pairing strategies for the Swedish, English, and Norwegian study samples. The red triangles represent the average screening performance for the random pairing strategy, the black dots represent the specific pairing strategies, and the blue triangle with point down represents the performance for individual reading. Error bars are 95% confidence intervals, obtained by bootstrap resampling (*n* = 1,000) and adjusted for 7 comparisons. The TPR and FPR of the individual simulation were statistically significantly lower compared with the TPR and FPR of the double reading pairing strategies in all 3 data sets. FPR, false-positive rate; TPR, true-positive rate.

The performance measures of the individual reading simulation were compared against the double reading performance measures for the different pairing strategies (Figure 8, Appendix Table A.1). The TPR and FPR of the individual simulation (Sweden: TPR: 60.46% [95% CI: 59.08%–61.83%], FPR: 3.50% [95% CI: 3.47%–3.54%], England: TPR: 78.72% [95% CI: 77.97%–79.48%], FPR: 4.17% [95% CI: 4.13%–4.21%], and Norway: TPR: 69.53% [95% CI: 68.68%–70.39%], FPR: 5.10% [95% CI: 5.06%–5.13%]) were statistically significantly lower compared with the TPR and FPR of all double reading pairing strategies in all 3 data sets (Sweden: TPR ≥64.72%, FPR: ≥4.50%, England: TPR ≥81.23%, FPR: ≥5.51%, Norway: TPR ≥78.83%, FPR: ≥8.03%).

## Discussion

As demonstrated in this study, modeling can overcome limitations inherent to using real-world data directly. Our logistic regression models allowed for the exploration of both individual and paired reader performance during screening mammogram interpretation exhaustively, evaluating the latter even for case readings that did not take place in the real world. The strong Pearson correlations between the model-predicted values and observed data suggest that these models can effectively capture single-reader performance and disagreement rates between paired readers. While our study focused on pairing strategies in the context of breast cancer screening, the approach may have potential for broader applications across diverse research fields. The ability to model and simulate scenarios opens opportunities to explore real-world data sets with more statistical power.

There might be some discrepancies between the predicted performance and actual outcomes if this were to be implemented in actual screening practice. However, we anticipate that the modeled performance in this study will closely approximate actual performance since individual TPR and FPR and case-negative disagreement rates showed robust associations (Pearson correlation >0.7) between model-predicted and observed data. Case-positive disagreement rates resulted in lower levels of association. This can be attributed to the relatively low number of positive cases per pair in the observed data, highlighting the challenge of assessing reader performance in the context of rare events. Nevertheless, the derived models allowed for the exploration of different pairing strategies, something that would not have been possible with sufficient statistical power in actual screening reading data sets. Of particular interest was the pairing strategy that emphasized the similarity in FPR between readers. This strategy, when compared with random pairing, consistently yielded a reduction in the number of FPs for all 3 study samples. This can be explained by the fact that similar FPR readers had a higher degree of concordance in negative assessments, leading to lower paired FPR values and thus lower grouped FPR, whereas random or opposite characteristic readers in a pair disagree more, which, in turn, led to higher FPR values based on our pairing rule in which discrepant readings were classified as positive readings. Constructing pairs of radiologists with similar FPR characteristics did not show a significant change in overall TPR. This suggests that strategically pairing radiologists with similar FPR characteristics may increase reading performance. Additional analyses (Figure A1) also showed that pairing readers with similar FPR resulted in a significantly lower RR, while CDR did not significantly change. Therefore, pairing readers with similar FPR characteristics, thereby reducing FPR/RR without significantly changing TPR/CDR, may offer a practical solution to reduce the number of unnecessary examinations forwarded to consensus or arbitration, most probably without negatively affecting CDRs in actual practice. Such a reduction will reduce the workload on health care professionals and the number of potential false-positive recalls, alleviating the burden on both screened women and the health care system.

The differences in performance introduced by the different pairing strategies are overall small, as could be expected,^12^ but for a large screening program with a single picture archiving and communication systems (PACS) system, so might be the costs, if any, of implementing a specific paired-reading strategy. Such a PACS system, from which readers can read mammograms from any location, provides opportunities for strategic pairing by implementing automated pairing algorithms. The automatic pairing algorithms could be based on the available radiologists and their regularly benchmarked performance metrics. Peer review and feedback sessions of discrepant examinations may help radiologists improve their screening performance and bring the number of discrepancies in their FP down, naturally employing our pairing strategy of pairing readers with similar FPR characteristics. However, before implementing and to ascertain cost-effectiveness, thorough analyses should be conducted.

Further studies should show how radiologists that are relatively similar in FPR but still somewhat different in TPR can be distinguished. This is crucial to avoid missing more cases of cancers. Further studies are also needed to assess the impact of pairing similar FPR readers on final screening outcomes after consensus/arbitration. In addition, it should be noted that pairing readers with similar characteristic results in higher variation in performance among different pairs. In contrast, pairing opposite characteristic readers results in more consistent performance measures across pairs, consequently leading to more consistent results among women. There may also be another optimal pairing strategy that further maximizes paired reading performance based on other factors. A previously published study by Gandomkar et al.^27^ proposed pairing radiologists with different cognitive eye-tracking metrics to optimize double reading. However, this study was performed with an enriched test set and investigated the performance of different pairs but not the group performance of all radiologists together. Furthermore, eye trackers are not, perhaps yet, used in screening programs. Eventually, prospective screening studies should be considered before implementing any pairing strategy in practice.

Our findings also raise possibilities for other future applications, like lung cancer screening. Even in the nonmedical environment, similar models could be used, for instance if double reading were ever used in airport bag screening. Also, in breast cancer screening programs with single reading, a new potential use of an optimal pairing strategy may be if the program incorporates AI as a second stand-alone reader. This would allow for the AI settings to be adjusted to the performance of the paired human reader to optimize this hybrid reading screening performance. This is especially important since our findings do suggest that double reading is an important mechanism to improve early detection of breast cancer (Figure 8), as previously shown.^6,28,29^ However, the individual modeling in this study is based on readers who in screening practice performed double reading. Readers who know they will be reading on their own will probably behave differently, thus warranting further research. In programs already using double reading, AI might eventually replace one of the readers. The human-AI combination would then again allow for the AI settings to be adjusted to the performance of the paired human reader to optimize the combination of the human reader and AI. Finally, using AI for decision support for radiologists may help to align the FPR of the readers, enhancing our pairing strategy, especially when both readers rely on AI in a similar manner. However, such an approach warrants thorough additional investigation and is deemed beyond the scope of our current study objectives.

Our study has several strengths and limitations. A major strength of this study is the inclusion of 3 different data sets, which allowed us to evaluate the effect of the pairing strategies in 3 different countries with different screening practices. The fact that the results for all 3 data sets appear similar strengthens our conclusions. Furthermore, the models of radiologists’ assessments developed in this study showed strong associations between the modeled and observed data and may thus be helpful for future modeling studies that include actual screening interpretation, in which each examination is read by either 1 or 2 radiologists. A limitation of our study was that the pairing strategies were based on the difference in performance between readers, but many different combinations of pairing strategies exist, and we did not maximize the pairing strategies to be as similar or opposite as possible as a group. Furthermore, consensus/arbitration assessments could not be modeled as we did not have information on the consensus/arbitration readers to develop a model of that screening interpretation stage. We also did not have information on which readers were blinded, so we could not control for these differences. Further research with a large radiologists’ cohort that includes more information on lesions, the radiologists’ experience levels, and characteristics of consensus and/or arbitration readers is needed to possibly identify other prospective selection criteria for optimally pairing readers.

In conclusion, this study showed the potential of logistic regression models to predict individual and paired reader performance during screening mammogram interpretation. With it, it was shown that strategic pairing of radiologists with similar FPR characteristics demonstrates promise in reducing false positives while preserving overall TPR. Telemedicine provides opportunities for strategic pairing, but the implementation of automated pairing algorithms and exploration of additional prospective selection criteria for double reading are essential steps in translating these findings into practice. Future research should also focus on the impact of consensus and arbitration decisions on reader pairing strategies.

## Supplemental Material

sj-docx-1-mdm-10.1177_0272989X241264572 – Supplemental material for Modeling Radiologists’ Assessments to Explore Pairing Strategies for Optimized Double Reading of Screening Mammograms Supplemental material, sj-docx-1-mdm-10.1177_0272989X241264572 for Modeling Radiologists’ Assessments to Explore Pairing Strategies for Optimized Double Reading of Screening Mammograms by Jessie J. J. Gommers, Craig K. Abbey, Fredrik Strand, Sian Taylor-Phillips, David J. Jenkinson, Marthe Larsen, Solveig Hofvind, Mireille J. M. Broeders and Ioannis Sechopoulos in Medical Decision Making
