# Supplementary material for: Modeling Radiologists’ Assessments to Explore Pairing Strategies for Optimized Double Reading of Screening Mammograms
Source: Med Decis Making. 2024 Jul 30;44(7):828–42. doi: 10.1177/0272989X241264572 (PMC11490068; doi:10.1177/0272989X241264572)
Supplement: sj-docx-1-mdm-10.1177_0272989X241264572 – Supplemental material for Modeling Radiologists’ Assessments to Explore Pairing Strategies for Optimized Double Reading of Screening Mammograms [file sj-docx-1-mdm-10.1177_0272989X241264572.docx]

# Appendix

**
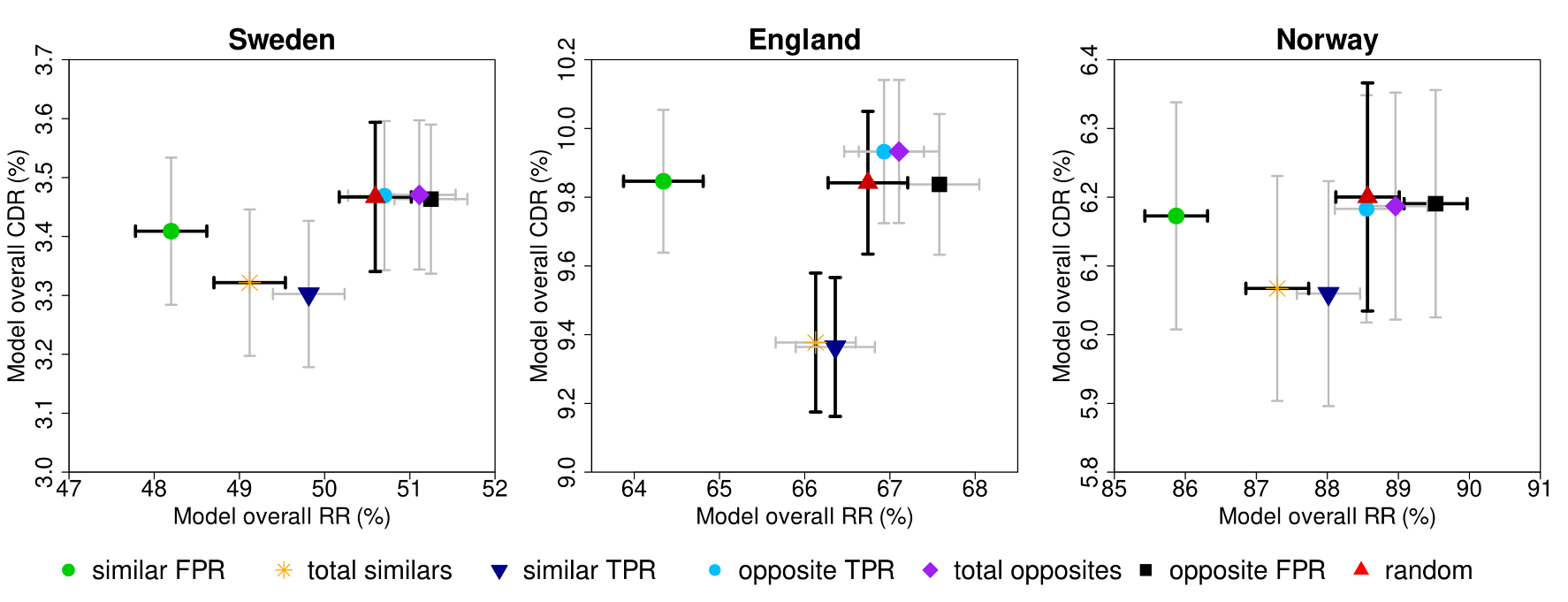
**To extrapolate the findings of Figure 6 to the context of screening programs, the resulting recall rate (RR) and cancer detection rate (CDR) of the different modelled pairing strategies were calculated. Figure A1 shows that the pattern of the RR and CDR graph looks similar to the pattern of the FPR and TPR graph (Figure 6). The confidence intervals indicate that pairing readers with similar FPR characteristics can significantly decrease RR without significantly affecting the CDR.

**Figure A1** – Screening performance for the different pairing strategies based on paired assessment.
The scatterplots show overall cancer-detection and recall rates of the different pairing strategies for the Swedish, English, and Norwegian study samples. The colors and symbols represent the different pairing strategies. Error bars are 95% confidence intervals, obtained by bootstrap resampling (n=1,000) and adjusted for 6 comparisons. Bold error bars indicate statistical significance. Please note that the axes are different, due to differences in CDR and RR between the study samples. CDR, Cancer-Detection Rate; RR, Recall Rate.

As a secondary analysis, the performance of individual reading was compared against that of double reading (Table A.1). Instead of comparing the 6 double reading pairing strategies against the random pairing strategy (Bonferroni adjustment 0.05/6), we now compared individual reading against all 7 double reading strategies (Bonferroni adjustment 0.05/7). Therefore, the confidence intervals in Table A.1 are slightly wider than those in Table 2 from the manuscript.

**Table A.1** – Screening performance for individual reading and double-reading pairing strategies

|  | Sweden | | England | | Norway | |
| --- | --- | --- | --- | --- | --- | --- |
|  | TPR (95% CI) | FPR (95% CI) | TPR (95% CI) | FPR (95% CI) | TPR (95% CI) | FPR (95% CI) |
| Similar FPR | 66.81  (65.45-68.16)* | 4.50  (4.46-4.54)* | 85.42  (84.76-86.08)* | 5.51  (5.47-5.56)* | 80.30  (79.55-81.05)* | 8.03  (7.99-8.07)* |
| Total similars | 65.10  (63.72-66.47)* | 4.60  (4.56-4.64)* | 81.35  (80.63-82.07)* | 5.74  (5.70-5.79)* | 78.93  (78.14-79.71)* | 8.19  (8.14-8.23)* |
| Similar TPR | 64.72  (63.34-66.10)* | 4.67  (4.63-4.72)* | 81.23  (80.51-81.96)* | 5.77  (5.72-5.81)* | 78.83  (78.05-79.62)* | 8.26  (8.22-8.30)* |
| Opposite TPR | 67.99  (66.66-69.31)* | 4.75  (4.71-4.79)* | 86.17  (85.53-86.80)* | 5.77  (5.72-5.81)* | 80.44  (79.69-81.18)* | 8.30  (8.26-8.34)* |
| Total opposites | 68.01  (66.69-69.34)* | 4.79  (4.75-4.83)* | 86.17  (85.54-86.80)* | 5.78  (5.74-5.83)* | 80.49  (79.74-81.23)* | 8.34  (8.30-8.38)* |
| Opposite FPR | 67.87  (66.53-69.22)* | 4.80  (4.76-4.84)* | 85.34  (84.69-85.99)* | 5.84  (5.80-5.89)* | 80.53  (79.78-81.28)* | 8.40  (8.36-8.44)* |
| Random | 67.95  (66.61-69.28)* | 4.74  (4.70-4.78)* | 85.38  (84.72-86.04)* | 5.76  (5.71-5.80)* | 80.66  (79.91-81.41)* | 8.30  (8.26-8.34)* |
| Individual (reference) | 60.46  (59.08-61.83) | 3.50  (3.47-3.54) | 78.72  (77.97-79.48) | 4.17  (4.13-4.21) | 69.53  (68.68-70.39) | 5.10  (5.06-5.13) |

TPR & FPR are percentages and 95% CI are Bonferroni adjusted (P-values <0.05/7) confidence intervals, obtained by bootstrap resampling (n=1,000). Non-overlapping confidence intervals with the individual reading strategy were regarded as statistically significant, as indicated by the asterisk. FPR, false-positive rate; TPR, true-positive rate.
